# BRCA1 mutation influences progesterone response in human benign mammary organoids

**DOI:** 10.1186/s13058-019-1214-0

**Published:** 2019-11-26

**Authors:** Batzaya Davaadelger, Mi-Ran Choi, Hari Singhal, Susan E. Clare, Seema A. Khan, J. Julie Kim

**Affiliations:** 1 Division of Reproductive Science in Medicine, Department of Obstetrics and Gynecology, Robert H. Lurie Comprehensive Cancer Center, Northwestern University Feinberg School of Medicine, 4-117, Chicago, IL 60611 USA; 2 Department of Surgery, Northwestern University Feinberg School of Medicine, Chicago, IL USA

**Keywords:** Breast organoid, BRCA1, Progesterone receptor, TPA

## Abstract

**Background:**

Women, who carry a germline BRCA1 gene mutation, have a markedly increased risk of developing breast cancer during their lifetime. While BRCA1 carriers frequently develop triple-negative, basal-like, aggressive breast tumors, hormone signaling is important in the genesis of BRCA1 mutant breast cancers. We investigated the hormone response in BRCA1-mutated benign breast tissue using an in vitro organoid system.

**Methods:**

Scaffold-free, multicellular human breast organoids generated from benign breast tissues from non-carrier or BRCA1 mutation carriers were treated in vitro with a stepwise menstrual cycle hormone regimen of estradiol (E2) and progesterone (P4) over the course of 28 days.

**Results:**

Breast organoids exhibited characteristics of the native breast tissue, including expression of hormone receptors, collagen production, and markers of luminal and basal epithelium, and stromal fibroblasts. RNA sequencing analysis revealed distinct gene expression in response to hormone treatment in the non-carrier and BRCA1-mutated organoids. The selective progesterone receptor modulator, telapristone acetate (TPA), was used to identify specifically PR regulated genes. Specifically, extracellular matrix organization genes were regulated by E2+P4+TPA in the BRCA1-mutated organoids but not in the non-carrier organoids. In contrast, in the non-carrier organoids, known PR target genes such as the cell cycle genes were inhibited by TPA.

**Conclusions:**

These data show that BRCA1 mutation influences hormone response and in particular PR activity which differs from that of non-carrier organoids. Our organoid model system revealed important insights into the role of PR in BRCA1-mutated benign breast cells and the critical paracrine actions that modify hormone receptor (HR)-negative cells. Further analysis of the molecular mechanism of BRCA1 and PR crosstalk is warranted using this model system.

## Background

Breast cancer is the most common cancer, and the second leading cause of cancer death among women in the USA. The National Cancer Institute (NCI) estimates there will be 268,600 new cases and an estimated 41,760 deaths from this disease in 2019 in the USA [1]. Approximately 12% of women will be diagnosed with breast cancer at some point during their lifetime [1]. Women who carry a germline mutation in the breast cancer-associated gene 1 (BRCA1) have an increased risk (60–85%) of developing early-onset breast cancer, often diagnosed in the 4th and 5th decades of life, with 50% of cancers occurring before age 40 [1, 2]. The mechanisms associated with breast carcinogenesis in the high-risk BRCA1-mutated (BRCA1^mut^) population are not completely understood.

The human mammary gland consists of lobules that are confined by a basement membrane and surrounded by loose intralobular connective tissue, consisting of fibroblasts, lymphocytes and plasma cells, macrophages, and blood vessels. Surrounding the large ducts and terminal duct lobular units is the interlobular stroma that is more dense and collagenous compared to intralobular stroma [3, 4]. In the functional lobular unit, the mammary duct epithelium is comprised of two main cell types which include an inner layer of secretory luminal cells and outer layer of basal/myoepithelial cells. Only 10–15% of luminal epithelial cells express the hormone receptors (HRs), estrogen receptor (ER), and progesterone receptor (PR) [5]. ER and PR in the luminal epithelial cells drive the biological changes of the breast in response to estrogen (E2) and progesterone (P4) during each menstrual cycle. The rise and fall of E2 and P4 during the cycle affect tissue growth and turnover of various cell types and extracellular matrix in the breast [6, 7]. The highest rate of proliferation occurs during the mid-luteal phase when P4 levels peak [7]. Thus, it is evident that hormone action in the mammary gland involves intricate paracrine actions between cells for such changes to occur throughout the breast.

A number of different cell types in the mammary gland are in active communication with each other and with the extracellular matrix (ECM). Hormone receptor activation and signaling in the mammary gland are mediated by both autocrine and paracrine actions. To date, there is a lack of human benign breast model systems that represent these features. Primary human breast epithelial cells grown as monolayers in vitro lose steroid receptor expression which limits the ability to study E2 and P4 action long term [8].

Although studies in breast cancer show an interaction between BRCA1 and PR leading to an inhibition of PR activity on gene expression and cell proliferation [9, 10], there is limited information on how BRCA1 influences PR signaling in benign breast tissue. Tumors that develop in BRCA1 mutation carriers are usually basal-like and triple negative, with no expression of ER, PR, or HER2 [11, 12]. Despite their negative receptor status, these tumors arise from a niche that is hormonally rich [13, 14]. Evidence that supports the role of hormone signaling in the genesis of BRCA1-mutated breast cancers includes studies demonstrating that bilateral prophylactic oophorectomy reduces the risk of mammary tumors in mice, with a mammary targeted deletion of BRCA1 [15]. Furthermore, the efficacy of a progesterone receptor antagonist mifepristone (RU486) has been shown to be effective in preventing mammary tumors in BRCA1 null mice [16]. The mechanisms by which hormones promote cancer development in BRCA1 mutation carriers remain to be investigated.

Our goal was to investigate hormone response in the human benign breast of BRCA1^mut^ and non-carriers in an in vitro system. Benign breast organoids generated from breast tissues of women undergoing surgery were treated with fluctuating levels of E2 and P4 that mimic a 28-day menstrual cycle. Organoids were also treated with the selective progesterone receptor modulator, telapristone acetate (TPA), in order to determine PR activity. RNA sequencing revealed distinct progesterone gene signatures in BRCA1-mutated breast organoids compared to breast organoids obtained from non-carriers.

## Materials and methods

### Generation of mammary organoids

Human benign breast tissue specimens were obtained from women undergoing reduction mammoplasty (non-carriers) or from prophylactic mastectomy specimens from BRCA1 mutation carriers at Prentice Women’s Hospital of Northwestern Medicine, following patient consent. This study was reviewed and approved by Northwestern’s Institutional Review Board. Information regarding the type of surgery, age, menopause status, and BRCA1 mutation type is provided in Additional file 8: Table S1.

Human breast organoids were prepared from breast tissues obtained at surgery using modifications to methods of Stampfer et al [17] and Gomm et al [18]. Briefly, breast tissue was cut in millimeter-sized pieces and digested enzymatically using Ham’s F-12K (Kaighn’s) medium using 1 mg/mL collagenase type I (Life Technology) with shaking for 16–20 h at 37 °C. The resulting micro-sized structures were separated into various sizes using cell strainers with 40-, 70-, and 100-μm mesh pores. Organoids between 40 and 100 μm were maintained in complete MammoCult medium with the provided proliferation supplement, in addition to heparin at a final concentration of 4 mg/mL and hydrocortisone at a final concentration of 0.48 mg/mL (StemCell Technologies) and 1% pen/strep (Sigma), and placed in ultra-low attachment plates (Corning Costar). The organoids were then frozen in stem cell media (ATCC). All mammary organoids used in this study were thawed from frozen stocks and plated in ultra-low attachment plates and maintained in the medium for 24–48 h prior to treatment with the hormones (E2+P4) and TPA. Media was changed every 2–3 days.

### Hormone treatments

A stepwise E2 and P4 (Sigma) hormone treatment of 28 days was used to treat the mammary organoids to mimic the menstrual cycle [19, 20]. The hormone treatment was 0.1 nM E2 (days 0–7), 1 nM E2 (days 7–14), 1 nM E2+10 nM P4 (days 14–21), and 0.1 nM E2+50 nM P4. For another group of organoids, 1 μM of TPA (Repros Therapeutics) was added during the last 14 days of the hormone treatment. Media was changed every 2–3 days. All hormones were dissolved in cell culture-grade ethanol and stored at − 20 °C.

### Histology and immunostaining

After the 28-day hormone treatments, the mammary organoids were collected and fixed in 4% paraformaldehyde for 2 h, washed in 50% and 70% ethanol, and transferred into the cap of an Eppendorf tube that was used as an embedding mold. A warm 1% agarose solution was then poured over the organoids and allowed to solidify. The organoid/agarose blocks were then processed, paraffin embedded, and sectioned to 5-μm-thick slices. Paraffin sections were then processed for hematoxylin and eosin (H&E), trichrome stain and immunofluorescence (IF), and immunohistochemistry (IHC). The primary antibodies used were ER (1:200, Santa Cruz SC71064), PR (1:200, DAKO, M3568), pan cytokeratin (panCK; 1:200, Abcam, ab7753), Cytokeratin 18 (CK18; 1:200, Abcam, 93741), vimentin (Vim; 1:200, Abcam, 92547), α smooth muscle actin (αSMA; 1:200, Novus Biologicals, 600531), Cytokeratin 5/6 (CK5/6; 1:200, Agilent Technologies, M723729-2), COL3A1 (Atlas antibodies, 1:200, HPA007583), COL1A1 (LS Bio, 1:400, LS-C343921), MMP10 (LS Bio, 1:400, LS-C118518), MMP1(LS Bio, 1:400, LS-B8645), Ki67 (Dako, 1:200, GA62661-2), E-cadherin (BD Biosciences, 1:200, 610181), ITGB4 (LS Bio, 1:200, LS-B3778), and ALDH1A1 isoform (LSBio, 1:400, LS-B10149). For IHC, EnVision™+ System-HRP (DAB) (DAKO, K4010 and K4006) and HRP/DAB (ABC) Detection IHC (Abcam, ab64261) kits were used according to the manufacturer’s instructions, and slides were mounted in Cytoseal™ XYL (Thermo Scientific, 8312-4). For immunofluorescence, Alexa Fluor® 488-conjugated goat anti rabbit (1:1000, Life Technologies, A11008) and Alexa Fluor® 594- conjugated donkey anti mouse (1:1000, Jackson ImmonoResearch, 115-586-072) were used, and slides were mounted in ProLong™ Gold Antifade Mountant (Invitrogen, P36930). Nuclei were counter stained with either hematoxylin for 30 s or 1 μM DAPI (Invitrogen, D1306) for 15 min. For collagen staining, a trichrome stain kit (Abcam, ab150686) was used according to the manufacturer’s instructions. Routine hematoxylin and eosin (H&E) staining was performed using Harris hematoxylin (Thermo Scientific) stain and eosin stain (VWR). IHC images were acquired using a Leica DM5000 B microscope (40 × 0.75 N.A. objective) equipped with a CCD Leica. Immunofluorescent images were acquired using the Nikon A1 confocal microscope at the Northwestern Microscopy and Imaging Core.

### RNA extraction and real-time PCR

RNA was isolated using Tri Reagent (Sigma Aldrich, 93289) and Direct-zol™ RNA MicroPrep (Zymo Research, R2060) according to the manufacturer’s protocol with an additional step of DNase I treatment to remove any contaminating DNA. First-strand cDNA synthesis was then performed using 500 ng of RNA and M-MLV reverse transcriptase (Life Technologies). Primer sequences are shown in Additional file 9: Table S2. Fold change values were calculated using the comparative Ct method using *GAPDH* as the housekeeping gene.

### RNA sequencing (RNA-seq)

RNA-seq was performed at the NUSeq Core Facility, Northwestern University, Chicago, IL. To generate RNA sequencing libraries, RNA quality was assessed with the Agilent Bioanalyzer 2100. Directional mRNA libraries were prepared using Illumina TruSeq mRNA Sample Preparation Kits per manufacturer’s instructions. Equimolar concentrations of each cDNA library were pooled and sequenced on the Illumina HiSeq500. The quality of DNA reads, in fastq format, was evaluated using FastQC. The analysis of RNA-seq data was performed by Artificial Intelligene (www.artificialintelligene.com), Intelligene Technologies. Briefly, short reads were aligned to the hg19 human genome using STAR [21]. Subsequently, cufflink packages were used to perform transcript assemblies [22]. Downstream differential gene expression calling on the reference and experimental groups of interest was performed using DESeq [23]. To perform clustering analyses on a group of samples, a union of all the genes and their expression RPKM values within that group was generated to build a read count matrix for the group of interest. Various unsupervised and other machine learning techniques were applied to this composite read count matrix of interest. Log2 (Fold change) = 0.5 was used for cutoff for the analyses. The sample-sample correlation heatmaps represent the correlation observed between any two samples. The sample-feature heatmaps represent the signal intensity of a feature for any given sample. ggplot2, heatmap.2, and Pheatmap packages in R were used to build various heatmaps. Functional analysis was performed using gene set enrichment analyses [24].

### Statistical analysis

All statistical analysis was performed using GraphPad Prism version 8.0 (GraphPad Software). Unpaired *t*-tests were performed when comparing groups. Paired *t*-test was used when comparing treatments (Fig. 4). All data represent the mean ± SEM of a minimum of three independent experiments and data were considered statistically significant if the *p* value was < 0.05. Statistical analysis was performed as described in the figure legends. All sample sizes and *p* values are reported in the figures and figure legends.

## Results

### Generation of benign mammary organoids in vitro

**Fig. 1:**
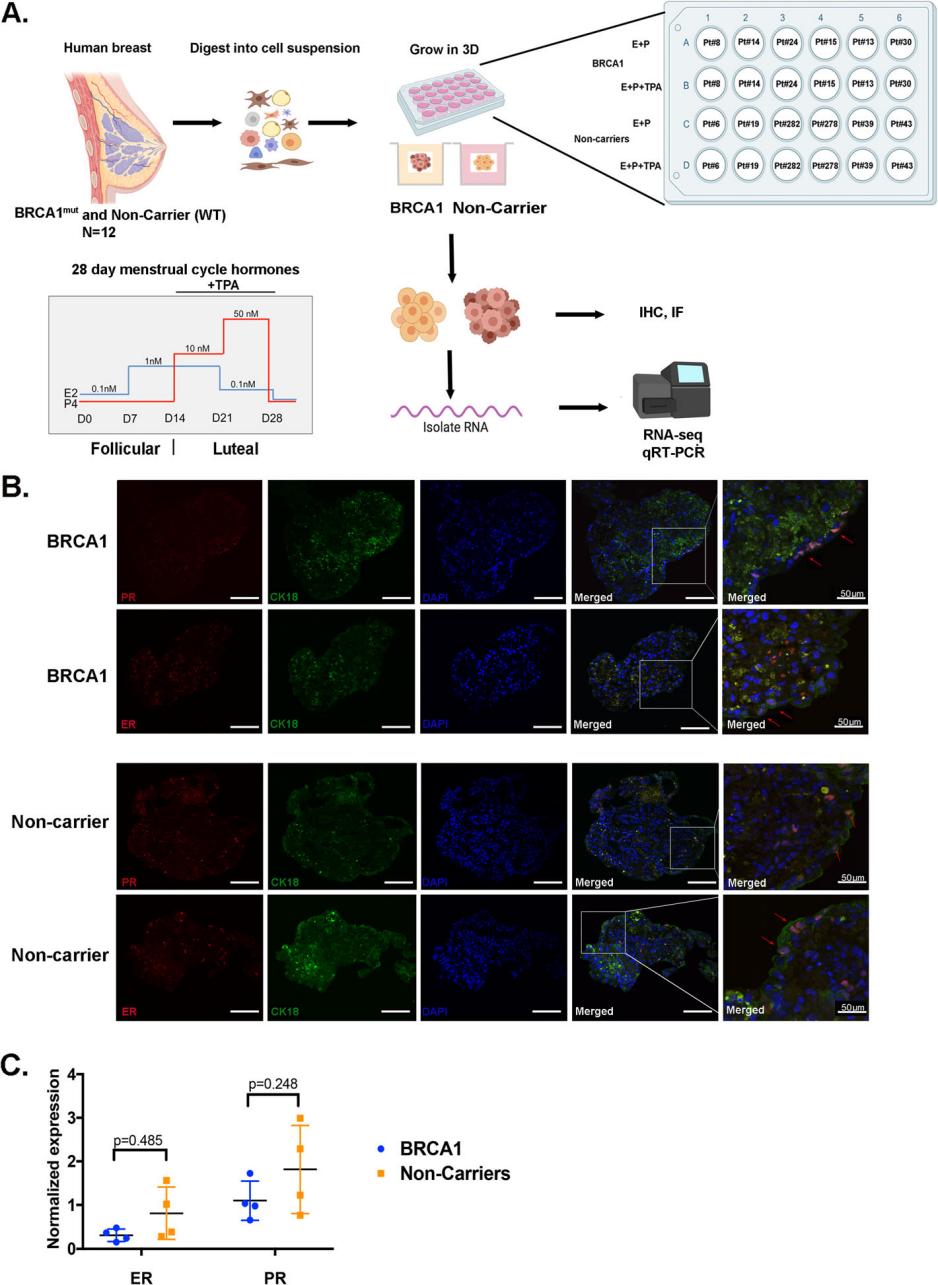
Generation of human mammary organoids. **a** Top, Schematic of overall study design is shown. Bottom panel is the 28-day stepwise menstrual cycle hormone profile. Day 0–day 14 Follicular phase, day 14–day 28 luteal phase. **b** PR and ER were co-stained with luminal marker (CK18) in BRCA1^mut^ and non-carrier organoids. Immunofluorescent staining was done for ER (red) and PR (red) along with CK18 (green) and DAPI (blue) to visualize the nuclei. Scale bar, 100 μm. Co-localization is shown by red arrows. **c** Normalized expression of ER and PR mRNA in BRCA1^mut^ and non-carrier breast organoids is shown. Unpaired *t*-test was performed

We generated an in vitro 3D organoid system using human benign breast tissues from patients undergoing reduction mammoplasty (RM) and prophylactic mastectomy from women with germline BRCA1 mutations (Fig. 1a). Detailed patient information is provided in Additional file 8: Table S1. Organoids derived from breast tissues from RM which are BRCA1 wildtype (WT) will be referred to as “non-carrier” and organoids derived from BRCA1-mutated tissues will be referred to as “BRCA1^mut^”. Individual patient-derived organoids are referred to as PT#. To mimic the human menstrual cycle, breast organoids were treated with a 28-day stepwise menstrual cycle hormone regimen replacing the medium every 2–3 days (Fig. 1a, bottom). During the last 14 days (luteal phase), when adding P4, telapristone acetate (TPA) was also added to identify PR target genes (Fig. 1a, bottom).

Immunofluorescent staining for ER and PR showed a focal subset of cells expressing ER or PR in both non-carrier and BRCA1^mut^ organoids (Fig. 1b). Co-staining with the luminal epithelial cell marker, CK18, showed co-localized with ER or PR (red arrows) but not with basal/myoepithelial cells (α smooth muscle actin, aSMA; Additional file 1: Figure S1). Negative controls for immunofluorescent staining are shown in Additional file 2: Figure S2.

As shown in Fig. 1c, all the patient-derived organoids expressed ER and PR mRNA at varying levels after the 28-day hormone regimen. These results demonstrate that mammary organoids grown in vitro in a scaffold-free 3D system for 28 days retain the expression of HRs which are expressed in a subset of luminal epithelial cells.

### Characterization of mammary organoids

**Fig. 2:**
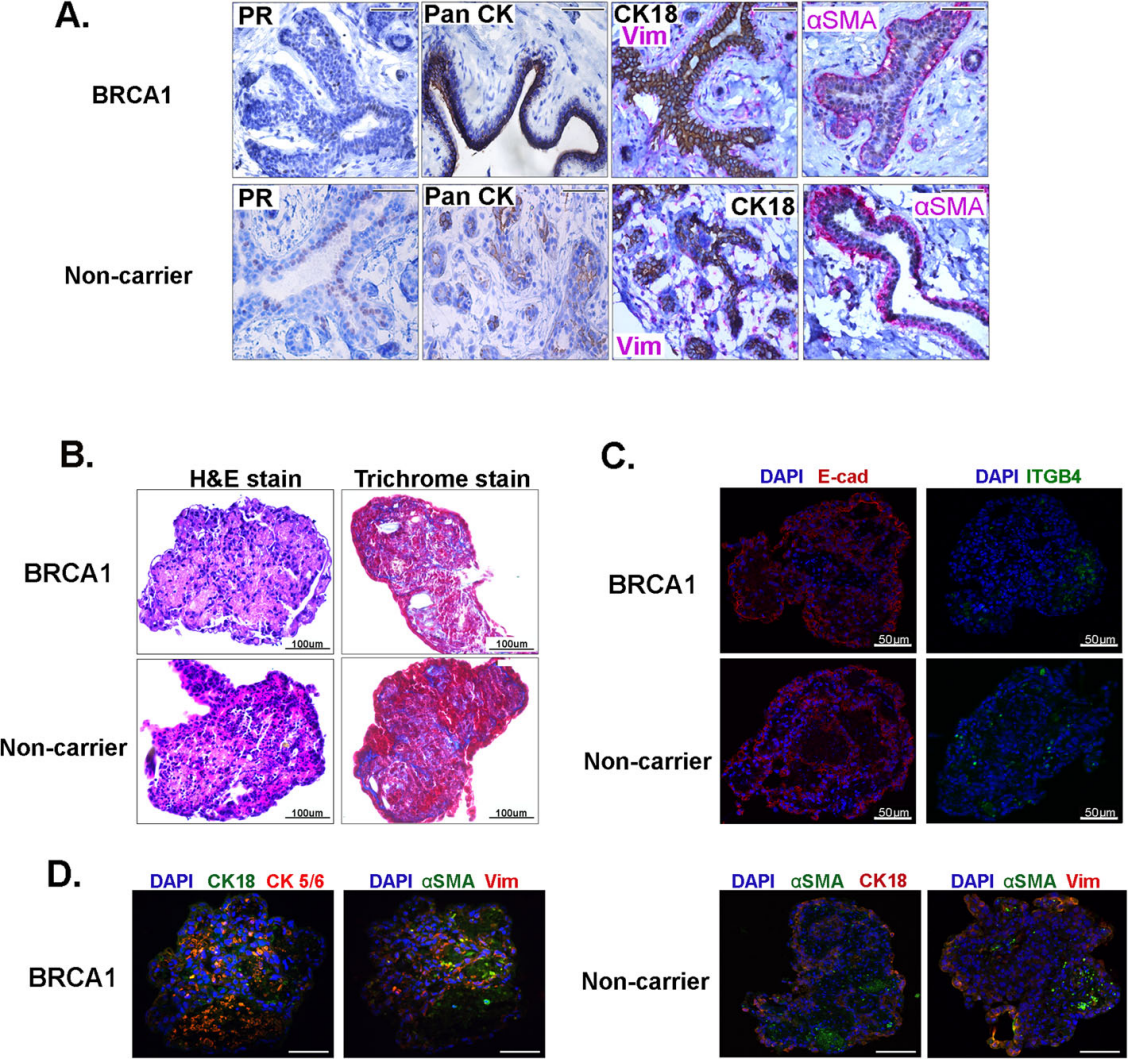
Characterization of mammary organoids. **a** IHC staining was done for PR (brown), epithelial marker pan CK (brown), luminal marker CK18 (brown), fibroblast marker vimentin (red), and basal marker αSMA (red) in non-carrier and BRCA1^mut^ breast tissue. Scale bar, 100 μm. **b** Left, H&E staining, Right, trichrome staining for collagen fibers (blue) is shown for BRCA1^mut^ and non-carrier organoids. **c** Immunofluorescent staining was done for E-cadherin (E-cad), red, a cell-cell adhesion marker and Integrin beta 4 (ITGB4), green, a cell-ECM adhesion marker in BRCA1^mut^ and non-carrier organoids. **d** Left, Immunofluorescent co-staining of BRCA1^mut^ organoid with luminal marker CK18 (red) and basal marker CK5/6 (green). Myoepithelial/basal marker αSMA (red) and fibroblast marker vimentin (green) is shown. Right panel is immunofluorescent co-staining of non-carrier organoids with luminal marker CK18 (red) and myoepithelial/basal marker αSMA (green), and myoepithelial/basal marker αSMA (red) and fibroblast marker vimentin (green). Scale bar, 100 μm

Immunofluorescent staining was done to visualize the architecture and composition of the breast organoids using specific cellular markers including pan cytokeratin (panCK; epithelial cell), CK18 (luminal epithelial cell), vimentin (Vim; fibroblast cell), and αSMA (basal/myoepithelial cell). As a comparative control, native breast tissues from non-carrier or BRCA1^mut^ patients were immunostained for the same markers. No distinct differences in cellular composition in the BRCA1^mut^ and non-carrier benign breast tissues were observed using these markers (Fig. 2a). Non-carrier and BRCA1^mut^ organoids both preserved extensive intercellular contacts and contained multiple cell types, shown by hematoxylin and eosin (H&E) staining (Fig. 2b). Trichrome staining which stains the collagen blue showed the presence of collagen within the BRCA1^mut^ and non-carrier organoids (Fig. 2b) suggesting that the fibroblasts are actively producing and depositing collagen to maintain the organoid structure. Proliferation of the organoids was assessed with Ki67 staining, and it was observed that at the end of the 28-day hormone treatment, some cells were proliferating (Additional file 3: Figure S3). In addition, immunofluorescent staining of E-cadherin, an epithelial cell-cell adhesion molecule, and Integrin (ITGB4), a protein that facilitates cell-ECM adhesion (Fig. 2c), were expressed in the BRCA1^mut^ and non-carrier organoids after the 28-day hormone cycle demonstrating intercellular contacts and communication. Furthermore, immunofluorescent staining for luminal, basal, and fibroblast markers (Fig. 2c, d) showed that the organoids contained multiple cell types. Together, these data show that BRCA1^mut^ and non-carrier organoids, derived from benign breast tissues preserve intercellular contacts, maintain an organoid structure and contain multiple cell types.

### Hormone response in non-carrier and BRCA1^mut^ organoids

**Fig. 3:**
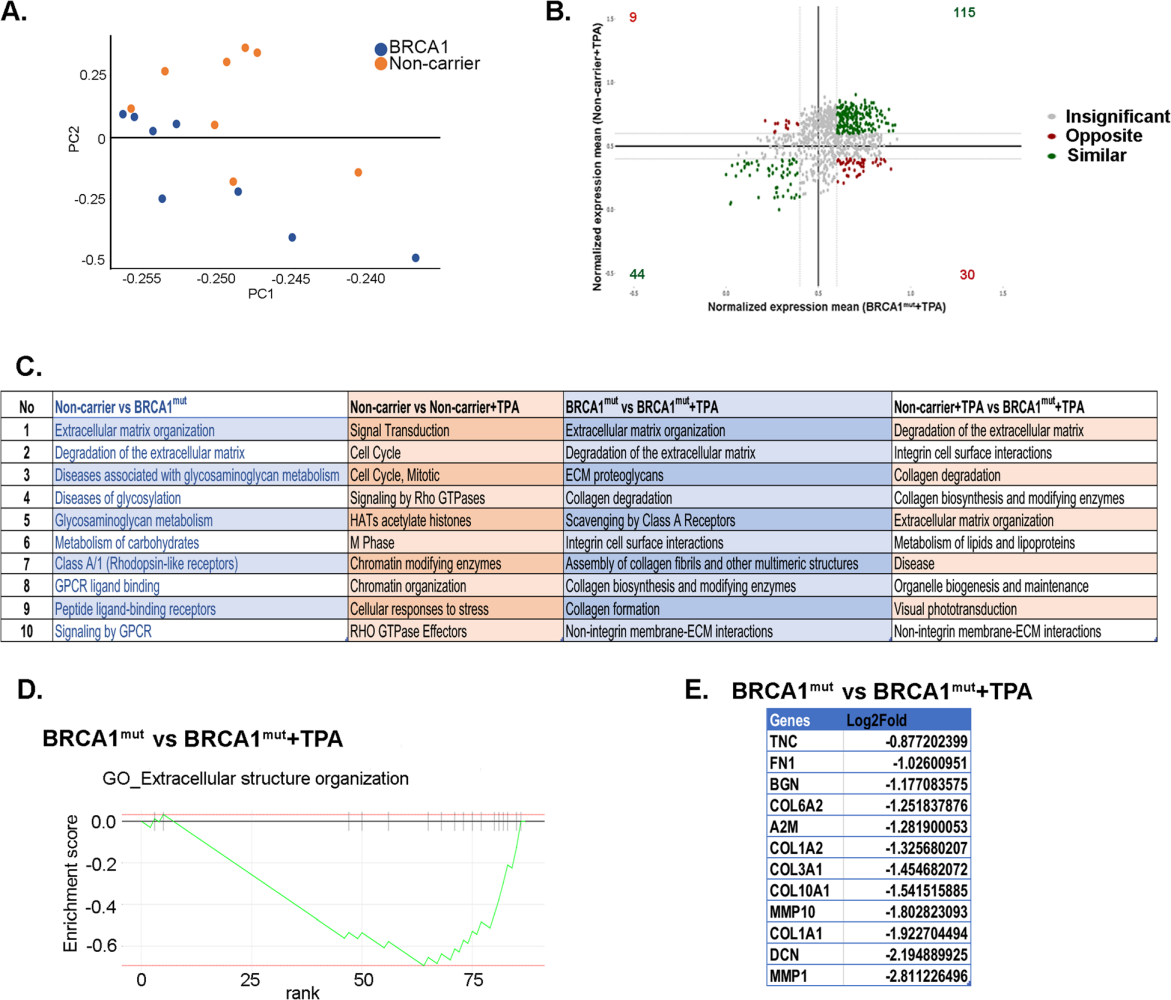
Differentially expressed genes in organoids from non-carrier and BRCA1-mutated tissues in response to hormones**. a** Principal component analysis (PCA) was done for the top 1% of variant genes in BRCA1^mut^ (*N* = 8, hormone and hormone+TPA) shown in blue, and in non-carriers (*N* = 8, hormone and hormone+TPA) shown in orange. **b** Correlation dot plot of expression of top 3% of variant genes between BRCA1^mut^+TPA (*N* = 4) and non-carriers+TPA (*N* = 4) is shown. **c** Gene ontology (GO) analysis was done for variant genes comparing different groups. *p* value *p < 0.05. **d** GSEA plot of extracellular matrix organization (*p* = 0.00012; NES = − 2.2987) shows the ECM organization signature is negatively enriched in BRCA1^mut^ tissues treated with E2+P4+TPA (*N* = 4) versus E2+P4 (*N* = 4). **e** Log2 fold change of extracellular organization genes in BRCA1^mut^ tissues treated with E2+P4+TPA (*N* = 4) versus E2+P4 (*N* = 4) is listed

It is unclear how PR functions in the background of BRCA1 mutations in non-cancerous benign mammary cells before breast cancer develops. In order to assess the hormonal response in the non-carriers and BRCA1^mut^ organoids at the transcriptomic level, RNA-seq was performed. Breast organoids from BRCA1^mut^ (*N* = 6) and non-carrier (*N* = 6) patients were treated with the 28-day stepwise menstrual cycle hormones, with or without TPA (Fig. 1a). RNA-seq data was analyzed using *Artificial Intelligene* (AI), an online integrated analysis system tool. Principal component analysis (PCA) plot showed BRCA^mut^ and non-carrier organoids clustering separately with expected heterogeneity among patient samples (Fig. 3a).

Four differential gene expression comparisons were done to assess the role of E2 and P4 in the non-carrier vs BRCA1^mut^, as well as the role of PR, specifically with the TPA for non-carrier and BRCA1^mut^ (non-carrier vs BRCA1^mut^, non-carrier vs non-carrier+TPA, BRCA1^mut^ vs BRCA1^mut^+TPA, and non-carrier+TPA vs BRCA1^mut^+TPA). Correlation of top 3% of most variant genes between the various treatment groups is shown in Fig. 3b and Additional file 4: Figure S4. The PR-regulated genes in the non-carrier+TPA versus BRCA1^mut^+TPA showed that 393 genes were equally expressed in both sample sets (Fig. 3b center, gray dots), 115 genes were highly expressed in both non-carrier TPA and BRCA1^mut^ TPA groups (top right, green), 9 genes had high expression in BRCA1mut+TPA group only (top left, red), 44 genes had low expression in both groups (bottom left, green), and 30 genes had low expression in BRCA1^mut^+TPA only (bottom right, red) (Fig. 3b). This data demonstrates that while some PR-responsive genes are similar between the two groups, there are genes that are differentially regulated in BRCA1^mut^ compare to non-carrier organoids.

When these genes were subjected to gene ontology analysis, distinct pathways were enriched between the non-carrier and BRCA1^mut^ groups (Fig. 3c and Additional file 10: Table S3). Genes involved in extracellular matrix (ECM) organization were enriched in response to menstrual cycle hormones (E2+P4) in BRCA1^mut^ compared to non-carrier organoids. In addition, these ECM-specific genes were differentially regulated by TPA in the BRCA1^mut^ group but not in the non-carrier group further suggesting different PR activity in BRCA1^mut^ organoids compared to non-carrier. Interestingly, known PR target genes that regulate the cell cycle were responsive to TPA in the non-carrier group only (Fig. 3c).

Gene set enrichment analysis (GSEA) showed that BRCA1^mut^ organoids treated with E2+P4+TPA exhibited a significant downregulation of ECM organization genes compared to the BRCA1^mut^ organoids treated with E2+P4 only (Fig. 3d). Shown in Fig. 3e, are the expression of specific ECM genes in E2+P4-treated BRCA1^mut^ organoids in response to TPA. Taken together, these data demonstrate that BRCA1 mutation influences hormone response and, in particular, ECM organization genes.

### Confirmation and validation of the effect on ECM genes

**Fig. 4:**
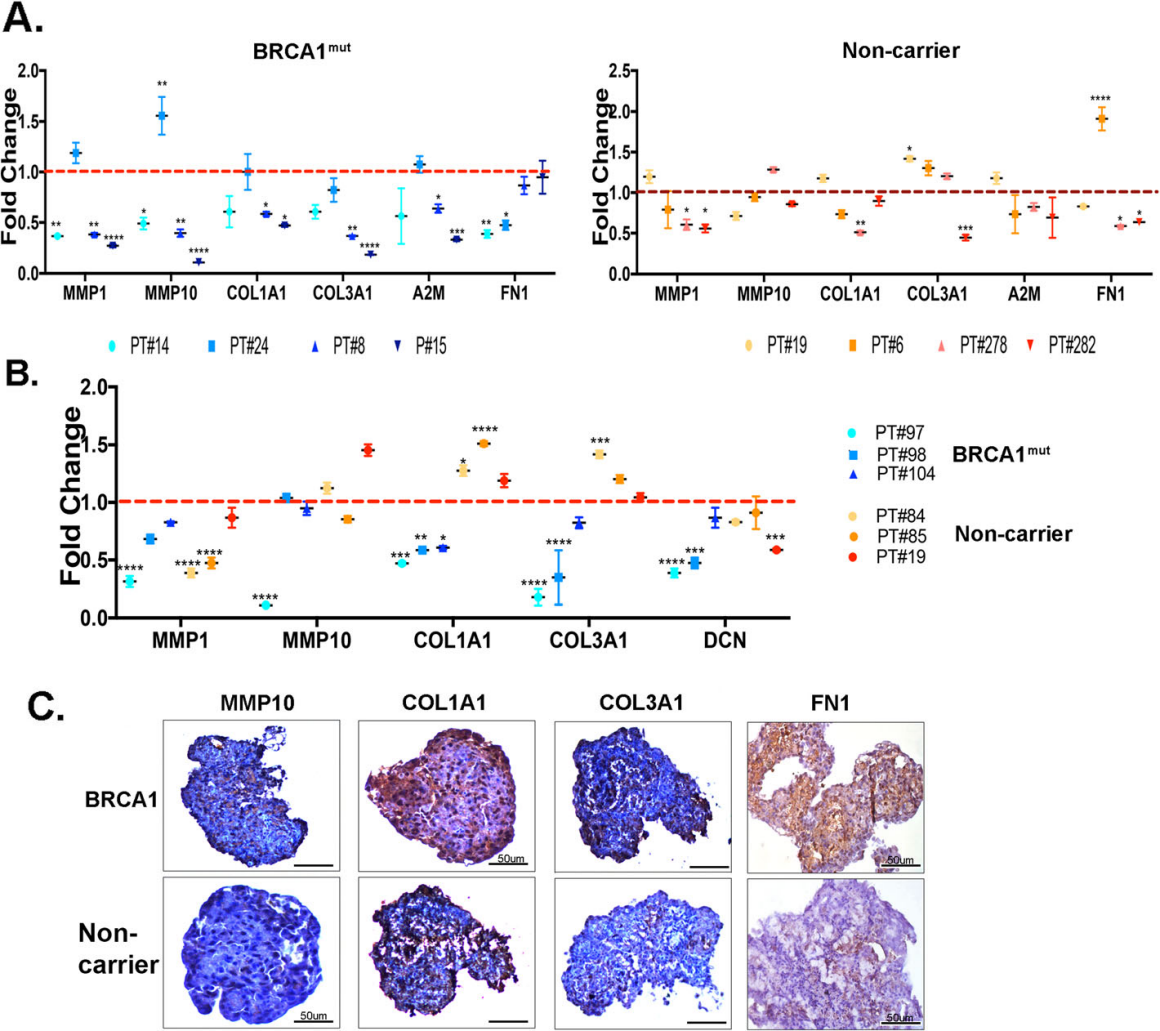
Confirmation and validation of ECM genes**. a** Confirmation of ECM genes was done by real-time PCR analysis of the samples used in the RNA-seq analysis. The data are fold changes of hormone treatment (dotted red line) and represent the mean ± SEM from three technical replicates (*p < 0.05, **p < 0.01, ***p < 0.005, ****p < 0.001). **b** Validation of ECM genes was done by real-time PCR using new patient-derived individual BRCA1^mut^ and non-carrier organoids treated with E2+P4 and E2+P4+TPA. The data are fold changes of hormone treatment (dotted red line) and represent the mean ± SEM from three technical replicates (*p < 0.05, **p < 0.01, ***p < 0.005, ****p < 0.001. **c** IHC staining of ECM proteins MMP10, COL1A1, COL3A1, and FN1 were performed in non-carrier and BRCA1^mut^ organoids. Scale bar, 100 μm

To confirm the RNA-seq data, quantitative real-time PCR was done for ECM genes in each patient-derived organoids from the RNA-seq samples referred to as PT#. The E2+P4+TPA data are represented as fold changes of E2+P4 treatment (Fig. 4a, dotted red line is at 1). As shown in Fig. 4a left, the ECM genes *MMP1*, *MMP10*, *COL1A1*, *COL3A1*, *A2M*, and *FN1* were significantly downregulated in individual BRCA1^mut^ organoids in response to TPA treatment. These genes were not downregulated with TPA in the non-carrier organoids with an exception of *MMP1*, *COL1A1*, and *FN1* genes in organoids P278 and P282 (Fig. 4a, right).

To validate our RNA-seq data, additional patient-derived organoids from BRCA1^mut^ and non-carriers were exposed to the same hormonal treatments (E2+P4) in the absence or presence of TPA. Similar to the RNA-seq confirmation data in Fig. 4a, ECM genes, *MMP1*, *MMP10*, COL1A1, COL3A1, and DCN from the new BRCA1^mut^ organoids (*N* = 3), were significantly downregulated by TPA, but not in the non-carrier organoids (*N* = 3) (Fig. 4b).

IHC staining of ECM proteins MMP10, COL1A1, COL3A1, and FN1 were performed in BRCA1^mut^ and non-carrier organoids treated with hormones (E2+P4) (Fig. 4c). Higher levels of the ECM proteins were observed in the hormone-treated BRCA1^mut^ compared to the non-carriers.

Taken together, these data validate our RNA-seq data and demonstrate that in the background of *BRCA1* mutation PR regulates ECM gene and protein expression.

### Identification of cell type marker genes in mammary organoids

**Fig. 5:**
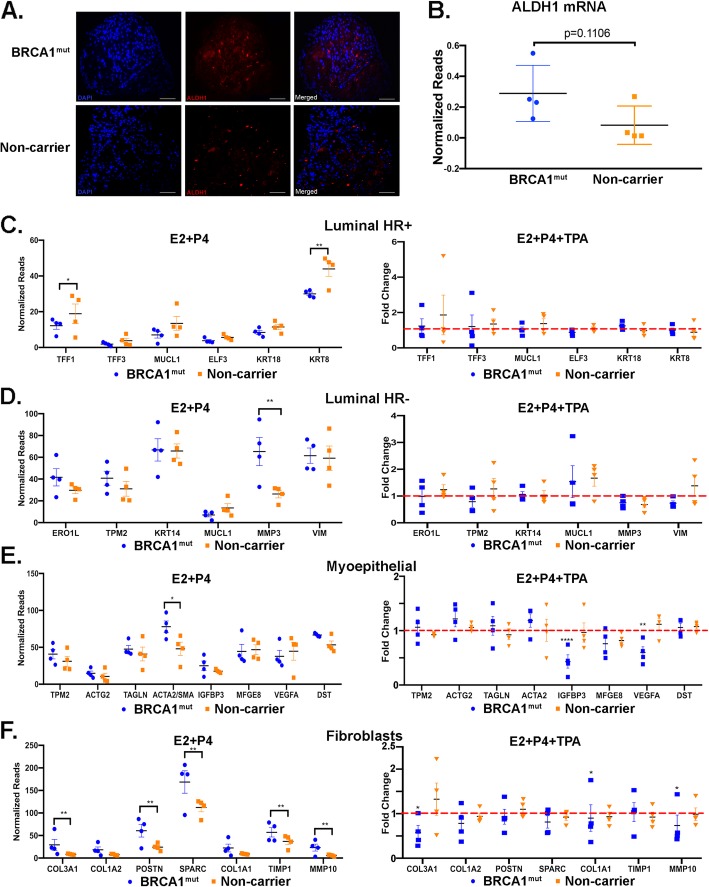
Expression of ALDH1 and cell type-specific genes in BRCA1^mut^ and non-carrier mammary organoids**. a** Immunofluorescent staining was done in BRCA1^mut^ and non-carrier organoids with ALDH1 (red) and DAPI (blue) to visualize the nuclei. Scale bar, 100 μm. **b** Normalized expression of ALDH1 mRNA in individual BRCA1^mut^ and non-carrier mammary organoids is shown (*N* = 4, *p* = 0.1106, unpaired *t*-test). **c–f** Normalized reads in individual BRCA1^mut^ and non-carrier organoids treated with E2+P4 are shown in the left panels for **c** luminal HR-positive genes, **d** luminal HR-negative genes, **e** myoepithelial genes, and **f** fibroblasts genes. The effect of TPA treatment in BRCA1^mut^ and non-carrier organoids are shown as fold changes of hormone treatment (red dotted line = 1) in the right panels for each cell-specific genes (*p < 0.05, **p < 0.01, ***p < 0.001)

RNA-seq data also demonstrated a differential regulation of the aldehyde dehydrogenase-1 (ALDH1) gene which has been shown to be a breast stem cell marker [25, 26]. Using immunofluorescent staining, we observed that ALDH1 protein levels were increased in the BRCA1^mut^ organoids treated with E2+P4 (Fig. 5a). Furthermore, the ALDH1 mRNA expression was increased in BRCA1^mut^ organoids compared to the non-carrier organoids (Fig. 5b). Interestingly, ALDH1 was present in the stroma of the organoids, which is consistent with a study showing the presence of ALDH1 in intralobular stroma and could be involved in breast stem cell renewal and differentiation [27].

The RNA-seq data was further mined to assess the expression of specific cell type markers in the BRCA1^mut^ and non-carrier mammary organoids. In a recent bioRxiv study, Murrow et al. performed single-cell (sc)-RNA sequencing in human breast tissues that were in the luteal and follicular phases of the menstrual cycle and identified seven different cell clusters [28]. The three major epithelial groups were hormone responsive luminal-hormone receptor positive (HR+), hormone-insensitive luminal-hormone receptor negative (HR−), and vascular accessory cells [28]. Each group had specific and distinct transcriptional profiles and differed depending on the menstrual cycle phase. Therefore, we looked at the expression of various genes specific to these cell types that were shown to be upregulated in the luteal phase. First, the expression of the luminal HR+ marker genes in hormone-treated non-carrier organoids were increased compared to hormone-treated BRCA1^mut^ organoids with *TFF1* and *KRT8* reaching statistical significance (Fig. 5c, left). When these marker genes were compared in BRCA1^mut^ and non-carrier organoids that were treated with TPA, the luminal HR+ marker genes were not significantly downregulated by TPA (Fig. 5c, right) which indicates that these genes are not regulated by PR. Second, the luminal HR-marker genes in hormone-treated BRCA1^mut^ and non-carrier organoids were analyzed (Fig. 5d, left). Luminal HR− marker genes were decreased in non-carrier hormone-treated organoids and *MMP3* was significantly downregulated compared to hormone-treated BRCA1^mut^ organoids. When we compared the luminal HR− marker genes in the TPA-treated BRCA1^mut^ and non-carrier groups, no significant downregulation by TPA was observed (Fig. 5d, right), again, suggesting that these genes are not PR target genes. Third, the myoepithelial (basal) marker genes in hormone-treated BRCA1^mut^ and non-carrier organoids were analyzed (Fig. 5e, left). Most of these marker genes were downregulated in hormone-treated non-carrier, and *ACTA2* was significantly downregulated compared to hormone-treated BRCA1^mut^ organoids. Interestingly, when we compared the myoepithelial marker genes in the TPA-treated BRCA1^mut^ and non-carrier groups, *IGFBP3* and *VEGFA* were significantly downregulated by TPA in BRCA1^mut^ organoids but not in the non-carrier organoids (Fig. 5e, right). This data suggests that *IGFBP3* and *VEGFA* are regulated by PR in the BRCA1^mut^ organoids through paracrine action but not in the non-carrier organoids. Finally, the fibroblast marker genes, mostly ECM genes in hormone-treated BRCA1^mut^ and non-carrier organoids were evaluated (Fig. 5f, left). As expected, most of the fibroblast marker genes were significantly increased in the hormone-treated BRCA1^mut^ organoids compared to the hormone-treated non-carrier organoids. Similarly, most of the fibroblast marker genes were significantly downregulated in response to TPA in hormone-treated BRCA1^mut^ organoids but not in the hormone-treated non-carrier organoids (Fig. 5f, right). Collectively, these data support that hormone responses and PR-regulated genes for certain breast cell type-specific genes are altered in BRCA1^mut^ organoids.

## Discussion

One of the impediments of understanding direct effects of risk factors on the breast is the lack of appropriate models that represent the in vivo human condition. Conventional cell cultures are usually monolayers of cancer cell lines and primary cells that are usually a single-cell type. While these systems are valuable in understanding cellular and molecular processes, they stop short of providing the necessary complexity that is required to represent human physiology. Multiple groups have studied human breast tissues ex vivo, to study hormone response, cell populations, and clonogenic potential. Graham and colleagues first demonstrated that human breast tissues embedded in Matrigel and grown in culture retained ER and PR expression and was responsive to progesterone [29]. Lim et al. demonstrated that FACS-sorted cells from human mammary tissues can be cultured as mammospheres [13]. They showed that breast tissues from *BRCA1*-mutated patients have an expanded luminal progenitor (LP) population and these cells showed higher clonogenic activity. A study by Proia et al. used a humanized mammary fat pad system to study breast tissues in vivo and reported that breast epithelial cells derived from women with a deleterious BRCA1 mutation gave rise to tumors resembling basal-like breast tumors [30]. Nolan et al. used an ex vivo 3D model using breast tissues from reduction mammoplasty (RM) that preserved extensive intercellular contacts and contained multiple cell types [31]. Each of these model systems has their own unique advantages and limitations, and data from the models should be interpreted accordingly, depending on the cell system and in vitro conditions. The unique features of our study include the multicellular and scaffold-free nature of the organoids from the human breast and the long-term survival with menstrual cycle hormones which provided a new understanding of hormone response in organoids from non-carriers and BRCA1 carriers. Specifically, organoid viability, expression of ER and PR, presence of multiple cell types, and cell proliferation occurred after 28 days of hormone treatment, and a significant difference in hormone response between BRCA1^mut^ and non-carrier organoids was observed. To determine how closely these organoids mimic the hormonal response of the native breast tissue, a parallel comparison with breast tissues biopsied from women at various stages of the menstrual cycle would be needed.

Within each organoid, a subset of cells expressed ER and PR which occurred mostly in luminal epithelial cells, yet the effects of hormones and TPA were observed in cells beyond the hormone receptor-positive cells highlighting the prominent paracrine actions of the breast. The relatively low expression of PR may have been due to the 14 days of progesterone treatment as it is known that progesterone downregulates PR [32]. The well-known PR mediators, RANKL, Wnt, and calcitonin [33], did not show a significant inhibition with TPA in our system in either the non-carrier or BRCA1^mut^ organoids (Additional file 5: Figure S5). Explanations for this could include the time-specific induction and activation of paracrine factors or the mixed population of cells each with different genomic signatures that would mask differential gene expression that occurs in a limited pool or cells. It is important to note that the changes in the organoids in response to the hormones are occurring throughout the 28 days and the cumulative effects at the end of the treatment time are measured. A finer analysis of the cell types and its paracrine factors by single-cell sequencing at specific time points of hormone treatment would reveal cell- and time-specific gene expression profiles.

The presence of multiple cell types in the organoids led to the discovery of the hormone effects in the BRCA1^mut^ organoids that extended into the stroma, as ECM genes were differentially regulated. The stroma produces various ECM proteins that not only provide mechanical support, but can also signal and influence other cell types in the mammary gland [34]. Our data strongly suggest that paracrine action between the cells may be dysregulated in BRCA1^mut^ breast tissues. Some of the ECM genes that were downregulated in BRCA1^mut^ organoids in response to TPA were MMPs, collagens, and fibronectins. MMPs are proteolytic enzymes that degrade structural components of the ECM, allowing remodeling to occur. A study by Radisky et al. showed that MMP-3 can cause epithelial to mesenchymal transition (EMT) and promote oxidative damage to DNA thereby inducing genomic instability and transformation in mouse mammary epithelial cells [35]. This new area of hormonal influence due to *BRCA1* mutation may be associated with increasing risk for breast cancer. In the clinic, mammographic density has been shown to be a risk factor for breast cancer [36]. A study by Mitchell et al. reported that high breast density in *BRCA* mutation carriers was associated with an increased risk of developing breast cancer [37]. A study by Huo et al. reported that *BRCA* mutation carriers tended to have more dense breast tissue than women from the general population [38]. The association between breast density and risk for breast cancer in BRCA carriers implicates the importance of ECM. Mechanistically, ECM stiffness can alter chromatin accessibility, which can promote the tumorigenic phenotype in mammary epithelium as supported by Stowers et al. [39]. They demonstrated that increased matrix stiffness displayed more accessible chromatin, which increased the binding of Sp1, a transcription factor that regulates the induction of stiffness-mediated tumorigenesis [39]. This could be a potential mechanism by which *BRCA1* mutation, which can affect matrix stiffness, could influence PR activity on chromatin. Further research is needed to address these mechanisms.

Other tumor-associated genes identified in our RNA-seq data which were differentially regulated in the BRCA1 organoids include NOTCH and ALDH1. Notch signaling has been implicated in the regulation of stem and progenitor cell fate determination as well as cell survival and proliferation [40]. The Notch signaling pathway interacts with BRCA1 in the breast via transcriptional upregulation of jagged 1 (JAG1) and regulates breast differentiation [41]. Our RNA-seq data revealed that Notch signaling was differentially regulated between non-carrier and BRCA1^mut^ organoids in response to hormones (Additional file 6: Figure S6). Although the differential regulation did not reach statistical significance due to patient variability, the hormone regulation of Notch signaling may be important in BRCA1-mutated breast tumorigenesis. We also observed an increased expression of ALDH1 in the BRCA1^mut^ organoids although TPA treatment did not change the ALDH1 levels. We have ruled out the possibility that the BRCA1-mutated tissues were naturally enriched with ALDH1-positive cells as immunohistochemical analysis of breast tissues from BRCA1^mut^ and non-carriers had similar levels of ALDH1 (Additional file 7: Figure S7). Ginestier and colleagues described ALDH1 as a marker for breast cancer stem cells [25]. Nolan and colleagues showed that ALDH1 was increased in the luminal progenitor (LP) cell population in BRCA1-mutated breast tissues [31]. Interestingly, Lim and colleagues isolated different cell types from breast tissue and found that the stromal fibroblasts expressed the highest levels of ALDH-1 compared to the epithelial cell population [13]. The increased levels of ALDH1 in both epithelial cells and stroma of the BRCA1 organoids in our study suggest an increased reservoir of potential stemness.

In order to understand why BRCA1 mutation carriers have an increased risk of developing breast cancer, it is important to study the benign breast with all of its complexities in a physiological manner. We have begun to study this using our multicellular breast organoid system and long-term menstrual cycle hormone treatments which have demonstrated differences in the breast tissues from BRCA1 carriers with non-carriers. New PR-regulated genes and pathways were discovered that provide a broader perspective of how hormones, with BRCA1 mutations, could increase risk of breast cancer.

## Conclusion

Our study is the first to investigate the effect of menstrual cycle hormones and a SPRM on BRCA1-mutated and non-carrier mammary organoids. Our physiologically relevant model system showed that PR regulation of genes differs in BRCA1 and non-carrier organoids. Furthermore, the changes observed in the ECM genes and others in BRCA1^mut^ organoids in response to hormones highlight the importance of paracrine actions to mediate the hormonal effects. Whether inhibiting these genes using SPRMs is an effective preventive measure in BRCA1 mutant tissues is an attractive possibility that requires further research.

## Supplementary information

**Additional file 1: Figure S1.** Immunofluorescent co-staining in BRCA1^mut^ and Non-carrier organoids. Immunofluorescent staining was done for ER (red) and PR (red) along with myoepithelial/basal marker αSMA (green) and DAPI (blue) to visualize the nuclei. Scale bar, 100 μm.

**Additional file 2:** **Figure S2.** Immunofluorescent negative controls. BRCA1^mut^ and Non-Carrier organoids were fluorescently stained with no primary antibody and Alexa-Fluor 555 (red) and 488 (green) secondary antibody and DAPI (blue) to visualize the nuclei. Scale bar, 100 μm.

**Additional file 3:** **Figure S3.** Ki67 staining. BRCA1^mut^ and Non-Carrier organoids were stained with Ki67 to measure proliferation.

**Additional file 4: Figure S4.** Correlation between top 3% most variant genes in comparison groups. Normalized expression mean of Non-carrier E2+P4 (*N*=4) vs BRCA1^mut^ E2+P4 (N=4), Non-carrier E+ P (N=4) vs Non-carrier+TPA (N=4), BRCA1^mut^ E2+P4 (N=4) vs BRCA1^mut^ + TPA (N=4).

**Additional file 5:** **Figure S5.** RANKL (TNFSF11) and Wnt4 mRNA expression. Normalized mRNA expression of TNFSF11 and Wnt4 in BRCA1^mut^ E2+P4 (N=4), BRCA1^mut^ E2+P4+TPA (N=4), Non-carriers E2+P4 (N=4) and Non-carriers E2+P4+TPA (N=4). Unpaired t-test was performed.

**Additional file 6: Figure S6.** Notch signaling GSEA plot. Notch signaling signature genes are positively enriched in the BRCA1^mut^ organoids treated with E2+P4 (N=4) versus Non-carrier organoids treated with E2+P4 (N=4). *p*=0.22, NES=1.23.

**Additional file 7:** **Figure S7.** ALDH1 staining. BRCA1^mut^ and Non-Carrier tissues were IHC stained with ALDH1. Scale bar, 100 μm.

**Additional file 8:** **Table S1.** Patient information.

**Additional file 9:** **Table S2.** qRT-PCR primer sequences.

**Additional file 10:** **Table S3.** Gene ontology analysis for genes differentially expressed in each of the four comparisons groups.

## Data Availability

The raw RNA-seq data have been deposited in the Gene Expression Omnibus database (www.ncbi.nlm.gov/geo) under accession number GSE131640.

## Abbreviations

ALDH-1: Aldehyde dehydrogenase-1
BRCA1: Breast cancer-associated gene 1
CK: Cytokeratin
E2: Estrogen
ECM: Extracellular matrix
EMT: Epithelial to mesenchymal transition
ER: Estrogen receptor
H&E: Hematoxylin and eosin
HR: Hormone receptor
IF: Immunofluorescence
IHC: Immunohistochemistry
NCI: National Cancer Institute
P4: Progesterone
PR: Progesterone receptor
RNA-seq: RNA sequencing
sc-RNA-seq: Single-cell RNA-seq
TPA: Telapristone acetate
Vim: Vimentin
WT: Wildtype
αSMA: α smooth muscle actin

## References

[1] Institute NC (2019). SEER Stat Fact Sheets: Breast Cancer.

[2] Kotsopoulos JSC, Narod SA (2017). Can we prevent BRCA1 associated breast cancer by RANKL inhibition?. Breast Cancer Res Treat.

[3] Fleming JM, Long EL, Ginsburg E, Gerscovich D, Meltzer PS, Vonderhaar BK (2008). Interlobular and intralobular mammary stroma: genotype may not reflect phenotype. BMC Cell Biol.

[4] Pellacani D, Tan S, Lefort S, Eaves CJ. Transcriptional regulation of normal human mammary cell heterogeneity and its perturbation in breast cancer. EMBO J. 2019;38(14):e100330. 10.15252/embj.2018100330 PMC6627240 31304632

[5] Clarke AH RB, Potten CS, Anderson E. Dissociation between steroid receptor expression and cell proliferation in the human breast. Cancer Res. 1997;57(22):4987–91. 9371488

[6] Ramakrishnan R, Khan SA, Badve S (2002). Morphological changes in breast tissue with menstrual cycle. Mod Pathol.

[7] Atashgaran V, Wrin J, Barry SC, Dasari P, Ingman WV (2016). Dissecting the biology of menstrual cycle-associated breast cancer risk. Front Oncol.

[8] Tanos T, Sflomos G, Echeverria PC, Ayyanan A, Gutierrez M, Delaloye JF (2013). Progesterone/RANKL is a major regulatory axis in the human breast. Sci Transl Med.

[9] Ma Y, Katiyar P, Jones LP, Fan S, Zhang Y, Furth PA (2006). The breast cancer susceptibility gene BRCA1 regulates progesterone receptor signaling in mammary epithelial cells. Mol Endocrinol.

[10] Katiyar P, Ma Y, Fan S, Pestell RG, Furth PA, Rosen EM (2006). Regulation of progesterone receptor signaling by BRCA1 in mammary cancer. Nucl Recept Signal.

[11] Romagnolo AP, Romagnolo DF, Selmin OI (2015). BRCA1 as target for breast cancer prevention and therapy. Anti Cancer Agents Med Chem.

[12] Turner N, Tutt A, Ashworth A (2004). Hallmarks of ‘BRCAness’ in sporadic cancers. Nat Rev Cancer.

[13] Lim E, Vaillant F, Wu D, Forrest NC, Pal B, Hart AH (2009). Aberrant luminal progenitors as the candidate target population for basal tumor development in BRCA1 mutation carriers. Nat Med.

[14] Molyneux G, Geyer FC, Magnay FA, McCarthy A, Kendrick H, Natrajan R (2010). BRCA1 basal-like breast cancers originate from luminal epithelial progenitors and not from basal stem cells. Cell Stem Cell.

[15] McCarthy A, Savage K, Gabriel A, Naceur C, Reis-Filho JS, Ashworth A (2007). A mouse model of basal-like breast carcinoma with metaplastic elements. J Pathol.

[16] Poole AJ, Li Y, Kim Y, Lin SC, Lee WH, Lee EY (2006). Prevention of Brca1-mediated mammary tumorigenesis in mice by a progesterone antagonist. Science..

[17] Stampfer M, Hallowes RC, Hackett AJ (1980). Growth of normal human mammary cells in culture. In Vitro.

[18] Gomm JJ, Browne PJ, Coope RC, Liu QY, Buluwela L, Coombes RC (1995). Isolation of pure populations of epithelial and myoepithelial cells from the normal human mammary gland using immunomagnetic separation with Dynabeads. Anal Biochem.

[19] Olalekan SA, Burdette JE, Getsios S, Woodruff TK, Kim JJ (2017). Development of a novel human recellularized endometrium that responds to a 28-day hormone treatment. Biol Reprod.

[20] Arslan SY, Yu Y, Burdette JE, Pavone ME, Hope TJ, Woodruff TK (2015). Novel three dimensional human endocervix cultures respond to 28-day hormone treatment. Endocrinology..

[21] Dobin A, Davis CA, Schlesinger F, Drenkow J, Zaleski C, Jha S (2013). STAR: ultrafast universal RNA-seq aligner. Bioinformatics..

[22] Trapnell C, Roberts A, Goff L, Pertea G, Kim D, Kelley DR (2012). Differential gene and transcript expression analysis of RNA-seq experiments with TopHat and cufflinks. Nat Protoc.

[23] Love MI, Huber W, Anders S (2014). Moderated estimation of fold change and dispersion for RNA-seq data with DESeq2. Genome Biol.

[24] Subramanian A, Tamayo P, Mootha VK, Mukherjee S, Ebert BL, Gillette MA (2005). Gene set enrichment analysis: a knowledge-based approach for interpreting genome-wide expression profiles. Proc Natl Acad Sci U S A.

[25] Ginestier C, Hur MH, Charafe-Jauffret E, Monville F, Dutcher J, Brown M (2007). ALDH1 is a marker of normal and malignant human mammary stem cells and a predictor of poor clinical outcome. Cell Stem Cell.

[26] Liu S, Ginestier C, Charafe-Jauffret E, Foco H, Kleer CG, Merajver SD (2008). BRCA1 regulates human mammary stem/progenitor cell fate. Proc Natl Acad Sci U S A.

[27] Kunju LP, Cookingham C, Toy KA, Chen W, Sabel MS, Kleer CG (2011). EZH2 and ALDH-1 mark breast epithelium at risk for breast cancer development. Mod Pathol.

[28] Lyndsay M, Murrow RJW, Caruso J, McGinnis CS, Borowsky AD, Desai TA, Thomson M, Tlsty T, Gartner ZJ. Mapping the complex paracrine response to hormones in the human breast at single-cell resolution. Cold Spring Harbor Laboratory-bioRxiv. 2018.

[29] Graham JD, Mote PA, Salagame U, van Dijk JH, Balleine RL, Huschtscha LI (2009). DNA replication licensing and progenitor numbers are increased by progesterone in normal human breast. Endocrinology..

[30] Proia TA, Keller PJ, Gupta PB, Klebba I, Jones AD, Sedic M (2011). Genetic predisposition directs breast cancer phenotype by dictating progenitor cell fate. Cell Stem Cell.

[31] Nolan E, Vaillant F, Branstetter D, Pal B, Giner G, Whitehead L (2016). RANK ligand as a potential target for breast cancer prevention in BRCA1-mutation carriers. Nat Med.

[32] Graham JD, Clarke CL (1997). Physiological action of progesterone in target tissues. Endocr Rev.

[33] Rajaram RD, Brisken C (2012). Paracrine signaling by progesterone. Mol Cell Endocrinol.

[34] Bissell MJ, Hall HG, Parry G (1982). How does the extracellular matrix direct gene expression?. J Theor Biol.

[35] Radisky DC, Levy DD, Littlepage LE, Liu H, Nelson CM, Fata JE (2005). Rac1b and reactive oxygen species mediate MMP-3-induced EMT and genomic instability. Nature..

[36] Vacek PM, Geller BM (2004). A prospective study of breast cancer risk using routine mammographic breast density measurements. Cancer Epidemiol Biomark Prev.

[37] Mitchell G, Antoniou AC, Warren R, Peock S, Brown J, Davies R (2006). Mammographic density and breast cancer risk in BRCA1 and BRCA2 mutation carriers. Cancer Res.

[38] Huo Z, Giger ML, Olopade OI, Wolverton DE, Weber BL, Metz CE (2002). Computerized analysis of digitized mammograms of BRCA1 and BRCA2 gene mutation carriers. Radiology..

[39] Stowers RS, Shcherbina A, Israeli J, Gruber JJ, Chang J, Nam S, et al. Matrix stiffness induces a tumorigenic phenotype in mammary epithelium through changes in chromatin accessibility. Nat Biomed Eng. 2019. 10.1038/s41551-019-0420-5 PMC6899165 31285581

[40] Dontu G, Jackson KW, McNicholas E, Kawamura MJ, Abdallah WM, Wicha MS (2004). Role of Notch signaling in cell-fate determination of human mammary stem/progenitor cells. Breast Cancer Res.

[41] Semmler L, Reiter-Brennan C, Klein A (2019). BRCA1 and breast cancer: a review of the underlying mechanisms resulting in the tissue-specific tumorigenesis in mutation carriers. J Breast Cancer.

