# Supplementary material for: BRCA1 mutation influences progesterone response in human benign mammary organoids
Source: Breast Cancer Res. 2019 Nov 26;21:124. doi: 10.1186/s13058-019-1214-0 (PMC6878650; doi:10.1186/s13058-019-1214-0)
Supplement: Supplementary file 1 — Additional file 1: Figure S1. Immunofluorescent co-staining in BRCA1mut and Non-carrier organoids. Immunofluorescent staining was done for ER (red) and PR (red) along with myoepithelial/basal marker αSMA (green) and DAPI (blue) to visualize the nuclei. Scale bar, 100 μm. [file 13058_2019_1214_MOESM1_ESM.pdf]

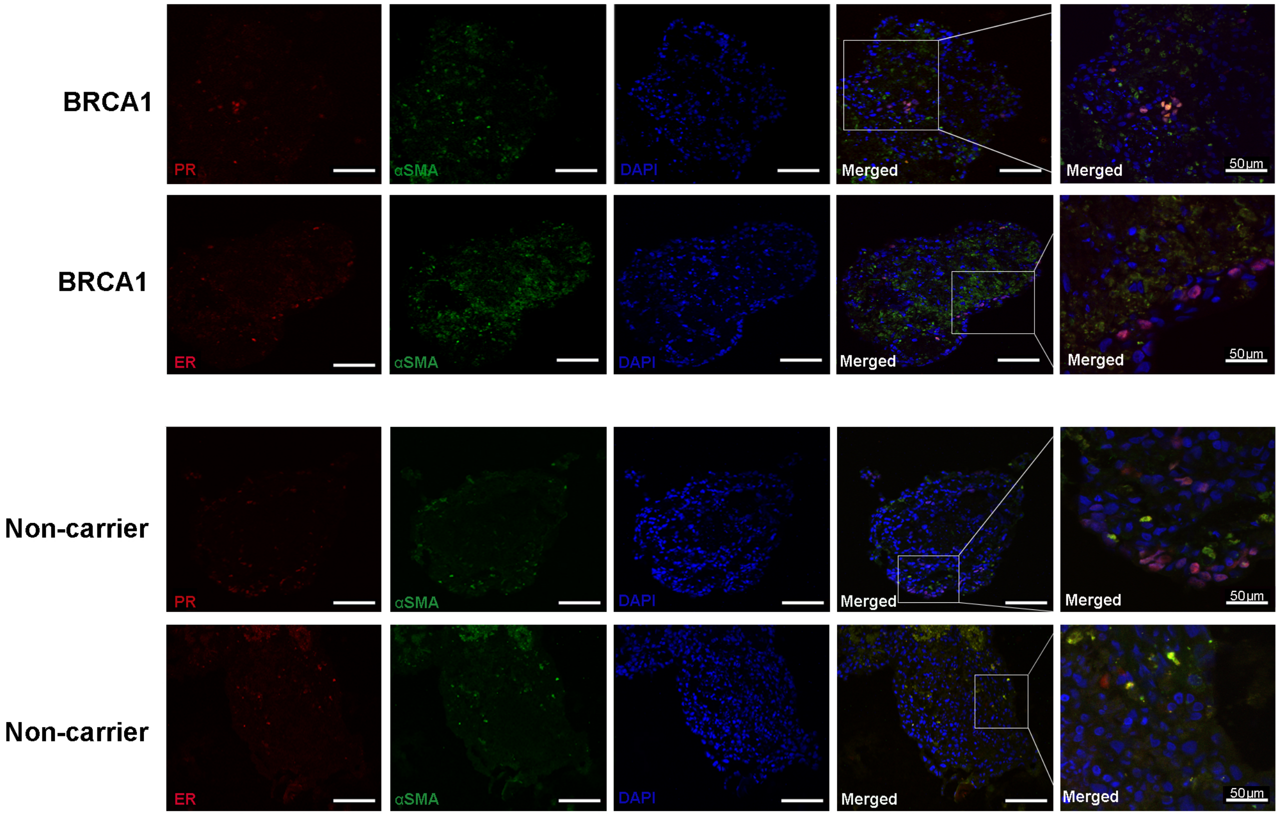

Supplemental Figure 1: Immunofluorescent co-staining in BRCA1^{mut} and Non-carrier organoids.

Immunofluorescent staining was done for ER (red) and PR (red) along with myoepithelial/basal marker αSMA (green) and DAPI (blue) to visualize the nuclei. Scale bar, 100 μm.
