# Supplementary material for: BRCA1 mutation influences progesterone response in human benign mammary organoids
Source: Breast Cancer Res. 2019 Nov 26;21:124. doi: 10.1186/s13058-019-1214-0 (PMC6878650; doi:10.1186/s13058-019-1214-0)
Supplement: Supplementary file 2 — Additional file 2: Figure S2. Immunofluorescent negative controls. BRCA1mut and Non-Carrier organoids were fluorescently stained with no primary antibody and Alexa-Fluor 555 (red) and 488 (green) secondary antibody and DAPI (blue) to visualize the nuclei. Scale bar, 100 μm. [file 13058_2019_1214_MOESM2_ESM.pdf]

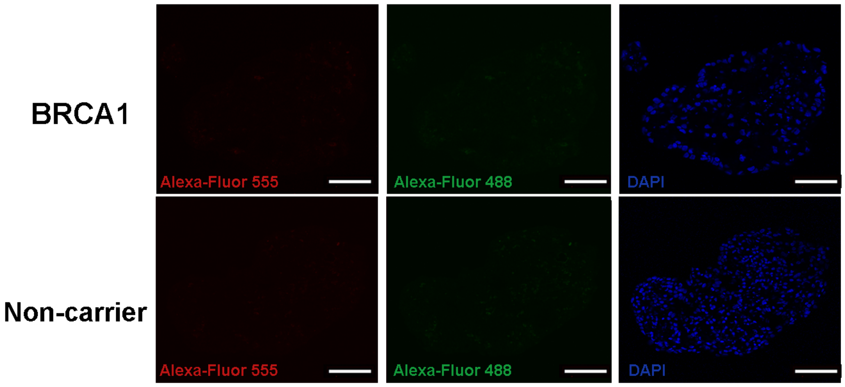

Supplemental Figure 2: Immunofluorescent negative controls.

BRCA1^{mut} and Non-Carrier organoids were fluorescently stained with no primary antibody and Alexa-Fluor 555 (red) and 488 (green) secondary antibody and DAPI (blue) to visualize the nuclei. Scale bar, 100 μm.
