# Supplementary material for: BRCA1 mutation influences progesterone response in human benign mammary organoids
Source: Breast Cancer Res. 2019 Nov 26;21:124. doi: 10.1186/s13058-019-1214-0 (PMC6878650; doi:10.1186/s13058-019-1214-0)
Supplement: Supplementary file 3 — Additional file 3: Figure S3. Ki67 staining. BRCA1mut and Non-Carrier organoids were stained with Ki67 to measure proliferation. [file 13058_2019_1214_MOESM3_ESM.pdf]

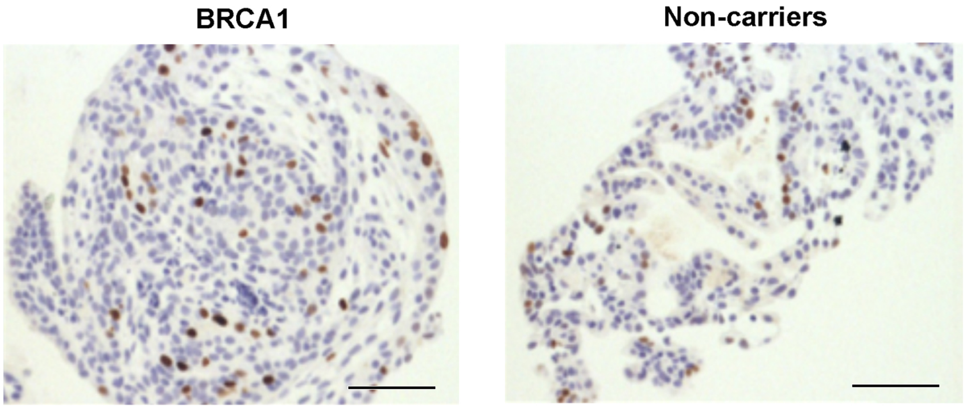

Supplemental Figure 3: Ki67 staining.

BRCA1^{mut} and Non-Carrier organoids were stained with Ki67 to measure proliferation.
