# Supplementary material for: BRCA1 mutation influences progesterone response in human benign mammary organoids
Source: Breast Cancer Res. 2019 Nov 26;21:124. doi: 10.1186/s13058-019-1214-0 (PMC6878650; doi:10.1186/s13058-019-1214-0)
Supplement: Supplementary file 4 — Additional file 4: Figure S4. Correlation between top 3% most variant genes in comparison groups. Normalized expression mean of Non-carrier E2+P4 (N=4) vs BRCA1mut E2+P4 (N=4), Non-carrier E+ P (N=4) vs Non-carrier+TPA (N=4), BRCA1mut E2+P4 (N=4) vs BRCA1mut + TPA (N=4). [file 13058_2019_1214_MOESM4_ESM.pdf]

Correlation between expression of selected genes

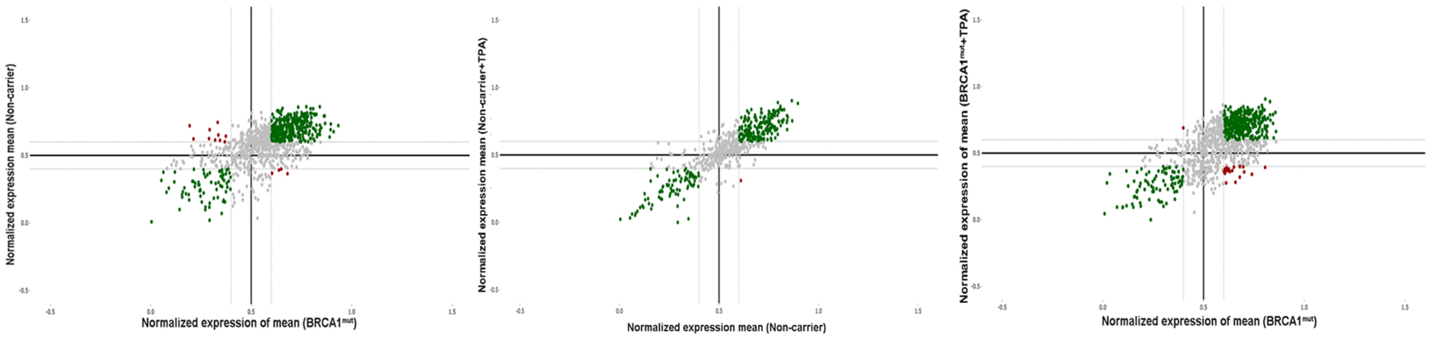

Supplemental Figure 4: Correlation between top 3% most variant genes in comparison groups.

Normalized expression mean of Non-carrier E+P (N=4) vs BRCA1^{mut} E+P (N=4), Non-carrier E+ P (N=4) vs Non-carrier+TPA (N=4), BRCA1^{mut} E+P (N=4) vs BRCA1^{mut} + TPA (N=4).
