# Supplementary material for: BRCA1 mutation influences progesterone response in human benign mammary organoids
Source: Breast Cancer Res. 2019 Nov 26;21:124. doi: 10.1186/s13058-019-1214-0 (PMC6878650; doi:10.1186/s13058-019-1214-0)
Supplement: Supplementary file 5 — Additional file 5: Figure S5. RANKL (TNFSF11) and Wnt4 mRNA expression. Normalized mRNA expression of TNFSF11 and Wnt4 in BRCA1mut E2+P4 (N=4), BRCA1mut E2+P4+TPA (N=4), Non-carriers E2+P4 (N=4) and Non-carriers E2+P4+TPA (N=4). Unpaired t-test was performed. [file 13058_2019_1214_MOESM5_ESM.pdf]

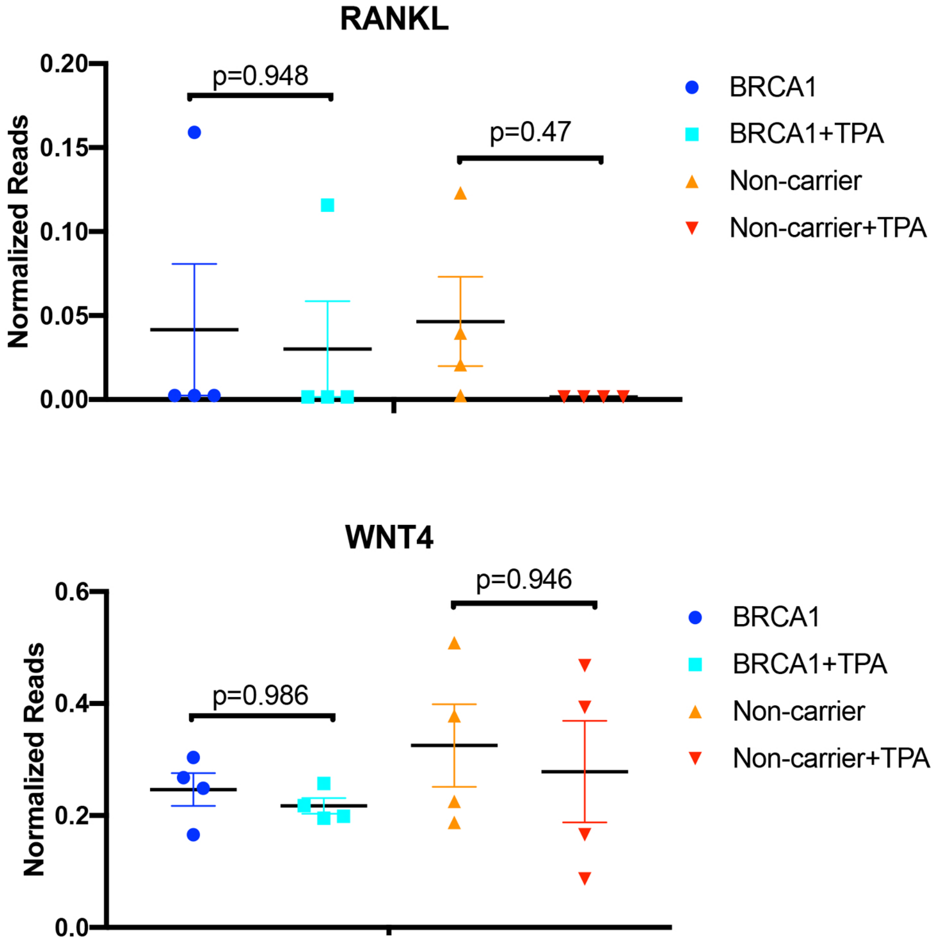

Supplemental Figure 5: RANKL (TNFSF11) and Wnt4 mRNA expression. Normalized mRNA expression of TNFSF11 and Wnt4 in BRCA1^{mut} E+P (N=4), BRCA1^{mut} E+P+TPA (N=4), Non-carriers E+P (N=4) and Non-carriers E+P+TPA (N=4). Unpaired t-test was performed.
