# Supplementary material for: BRCA1 mutation influences progesterone response in human benign mammary organoids
Source: Breast Cancer Res. 2019 Nov 26;21:124. doi: 10.1186/s13058-019-1214-0 (PMC6878650; doi:10.1186/s13058-019-1214-0)
Supplement: Supplementary file 6 — Additional file 6: Figure S6. Notch signaling GSEA plot. Notch signaling signature genes are positively enriched in the BRCA1mut organoids treated with E2+P4 (N=4) versus Non-carrier organoids treated with E2+P4 (N=4). p=0.22, NES=1.23. [file 13058_2019_1214_MOESM6_ESM.pdf]

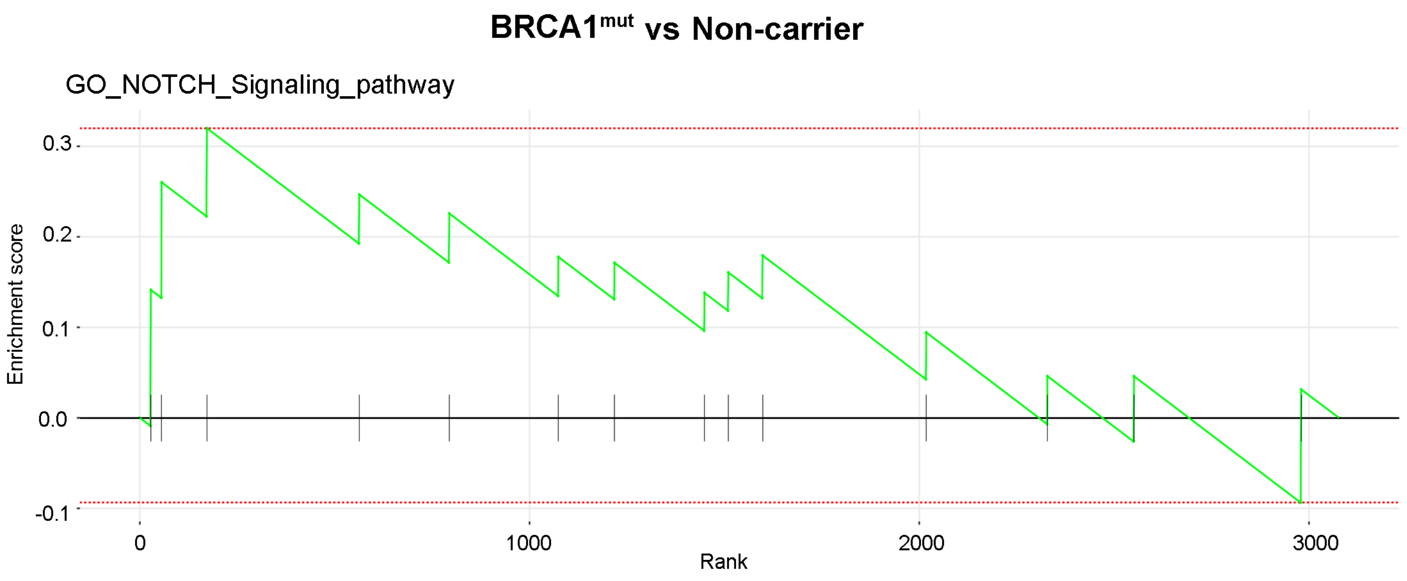

Supplemental Figure 6: Notch signaling GSEA plot.

Notch signaling signature genes are positively enriched in the BRCA1^{mut} organoids treated with E2+P4 (N=4) versus Non-carrier organoids treated with E2+P4 (N=4). p=0.22, NES=1.23
