# Supplementary material for: BRCA1 mutation influences progesterone response in human benign mammary organoids
Source: Breast Cancer Res. 2019 Nov 26;21:124. doi: 10.1186/s13058-019-1214-0 (PMC6878650; doi:10.1186/s13058-019-1214-0)
Supplement: Supplementary file 7 — Additional file 7: Figure S7. ALDH1 staining. BRCA1mut and Non-Carrier tissues were IHC stained with ALDH1. Scale bar, 100 μm. [file 13058_2019_1214_MOESM7_ESM.pdf]

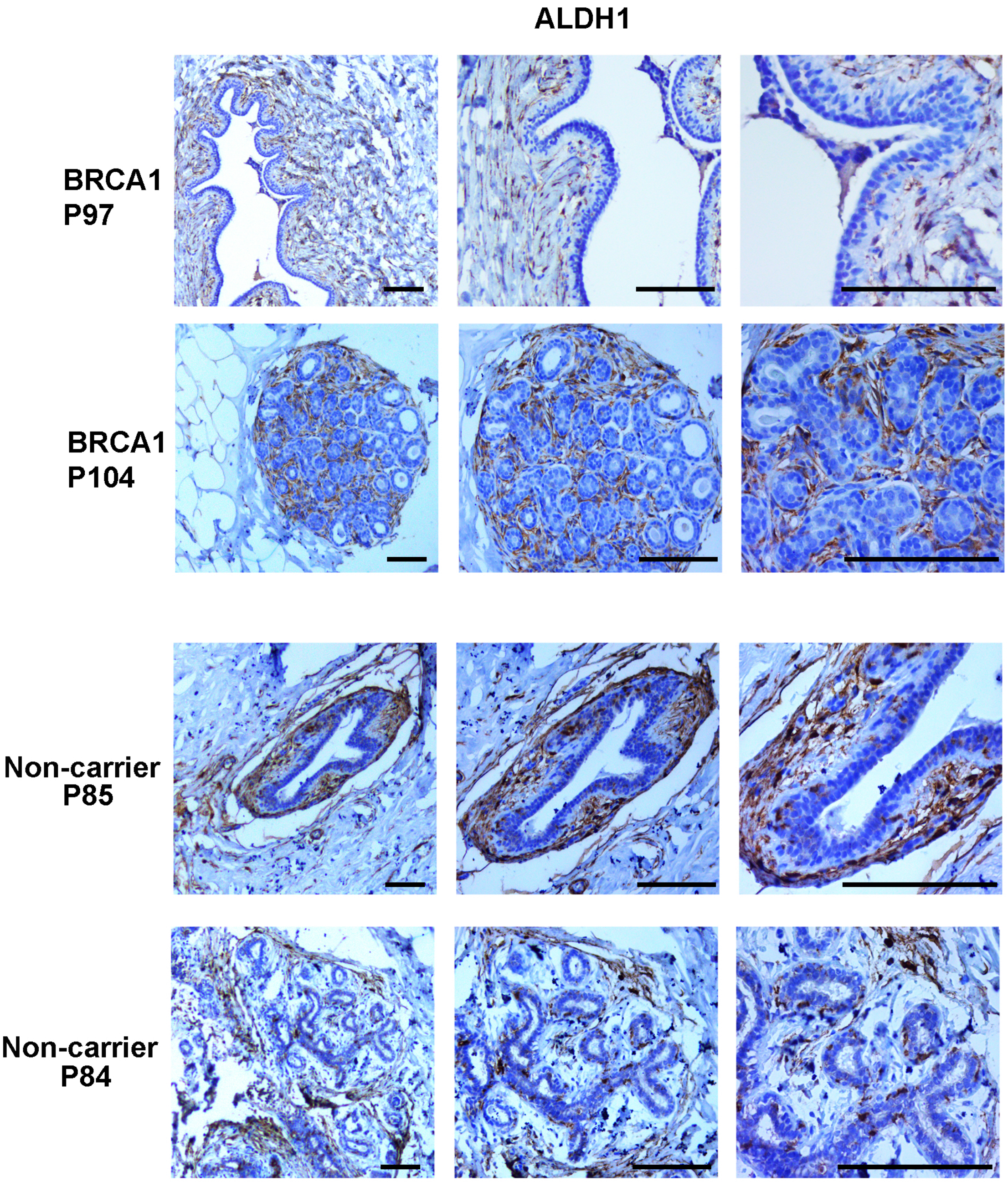

Supplemental Figure 7: ALDH1 staining. BRCA1^{mut} and Non-Carrier tissues were IHC stained with ALDH1. Scale bar, 100 μm.
